# Serotonergic and Cholinergic Imbalance in the Offspring of Rats Exposed to Bisphenol A and Bisphenol S During Pregnancy and Lactation: Short- and Long-Term Effects

**DOI:** 10.3390/ijms26199329

**Published:** 2025-09-24

**Authors:** Keila A. Semeão, Ana Carolina Dutra-Tavares, Anderson Ribeiro-Carvalho, Jemima Isnardo-Fernandes, Letycia D. Lopes, Gabriel S. M. Souza, André L. Nunes-Freitas, Beatriz S. Silva, Claudio C. Filgueiras, Alex C. Manhães, Patricia C. Lisboa, Yael Abreu-Villaça

**Affiliations:** 1 Laboratório de Neurofisiologia, Departamento de Ciências Fisiológicas, Instituto de Biologia Roberto Alcantara Gomes, Universidade do Estado do Rio de Janeiro (UERJ), Rio de Janeiro 20550-170, RJ, Brazil; brainherkeila@gmail.com (K.A.S.); jemimaisnardo@yahoo.com.br (J.I.-F.); dionisioletycia@gmail.com (L.D.L.); gabrielmartins1.1@hotmail.com (G.S.M.S.); andrefreitas01@gmail.com (A.L.N.-F.); ccfilg@yahoo.com.br (C.C.F.); ac_manhaes@yahoo.com.br (A.C.M.); 2 Departamento de Ciências Biomédicas e Saúde, Instituto de Biologia Roberto Alcantara Gomes, Universidade do Estado do Rio de Janeiro (UERJ), Cabo Frio 28905-320, RJ, Brazil; 3 Departamento de Ciências, Faculdade de Formação de Professores, Universidade do Estado do Rio de Janeiro (UERJ), São Gonçalo 20550-900, RJ, Brazil; ribeiro_carvalho@yahoo.com.br; 4 Laboratório de Fisiologia Endócrina, Departamento de Ciências Fisiológicas, Instituto de Biologia Roberto Alcantara Gomes, Universidade do Estado do Rio de Janeiro (UERJ), Rio de Janeiro 20550-170, RJ, Brazil; biabss1@gmail.com (B.S.S.); pclisboa@uerj.br (P.C.L.)

**Keywords:** sex differences, gestation, breastfeeding, depression, anxiety, cognition, programming, estrogen receptors

## Abstract

Considering the increased risk of cognitive deficits and mood disorders programming associated with bisphenol exposure, we used a preclinical model to identify short- and long-term effects of early exposure to Bisphenol A (BPA) and its replacement, Bisphenol S (BPS), on the central cholinergic and serotonergic systems. Wistar female and male rats born to dams exposed to BPA or BPS (both at 10 μg/kg/day or 50 μg/kg/day) during pregnancy and lactation were euthanized at weaning or adulthood. Cholinergic and serotonergic biomarkers were assessed in the frontal cortex and pons + medulla oblongata. BPA and BPS disrupted these systems, with outcomes depending on the specific bisphenol, biomarker, and time point. Effects also varied across brain regions and between sexes. The nicotinic cholinergic receptor showed more pronounced alterations than the presynaptic choline transporter. Both serotonergic receptors—5-HT1AR and 5-HT2R—were affected; however, the serotonergic transporter remained unchanged. Increased binding was the predominant effect for both systems. Maternal exposure to BPA, even at low doses, induces sex-dependent short- and long-term changes in the cholinergic and serotonergic systems of the progeny. BPS affects these same neurotransmitter systems, although leading to compound-specific outcomes. These results pose both BPA and BPS as neurotoxicants that compromise neurodevelopment and program disorders later in life.

## 1. Introduction

Endocrine-disrupting chemicals are natural or synthesized chemicals that mimic or interfere with the endocrine system. Among endocrine-disrupting chemicals, bisphenol compounds stand out as a ubiquitous group of environmental pollutants, used in the production of food cans and plastic bottles, flame retardants, dental sealants, antioxidants, and other products [1,2]. Exposure to bisphenol occurs through dietary and non-dietary sources [3,4] and, during sensitive periods such as pregnancy and lactation, can impact brain development in the offspring [3].

Bisphenol A (BPA) is the most studied component of this group of industrial chemicals. It is known to cross the placental and blood–brain barriers and is present in breastfeeding milk [5,6,7]. This bisphenol is considered a weak environmental estrogen due to its low binding affinity for estrogen receptors. As a result, its effects at low concentrations are thought to occur, in great part, through non-classical pathways, rather than through traditional nuclear or genomic estrogen receptor signaling [8]. On this matter, effects of BPA on other hormonal systems include its antiandrogenic activity [9,10], disrupted thyroid hormone function [11,12] and glucocorticoid-regulated responses [13].

Developmental exposure to BPA significantly impacts the immature brain [14,15], disrupting neurodevelopmental processes, which may manifest as mood disorders and cognitive deficits in the long term [15,16]. While the underlying mechanisms remain poorly understood, BPA targets are not restricted to the endocrine system [16]. Brain regions, including the frontal cortex and pons + medulla oblongata, are likely implicated in the establishment of impaired behaviors [17,18] and, within these regions, altered function of a number of neurotransmitter systems may contribute to the outcomes. In this regard, despite the established role of the serotonergic and cholinergic systems on mood and cognition [19,20,21,22], their involvement in BPA-related effects has yet to be thoroughly investigated. Current evidence provides only a fragmented view of alterations. Analysis of the serotonergic system in the raphe nuclei indicates that BPA exposure during gestation and lactation increases the number of serotonergic neurons in males, while, in females, it enhances serotonergic fiber density and dendritic branching [23]. The few other available studies report decreased expression of tryptophan hydroxylase type 2, the rate-limiting enzyme for serotonin synthesis, in the prefrontal cortex of adult male rats [24], as well as increased serotonin turnover and elevated mRNA expression of monoamine oxidase A in the pons and medulla oblongata of juvenile females [25]. To the best of our knowledge, only one study investigated serotonergic receptors, in which 5-HT_1A_R mRNA was reported elevated also in the pons and medulla oblongata of juvenile females [25]. The effects of perinatal BPA exposure on the cholinergic system have been even less extensively studied. Reduced levels of acetylcholine and acetylcholinesterase were reported in the prefrontal cortex and hippocampus of adult male rats [26], and, more recently, decreased choline levels were observed in the frontal cortex and cerebellum of near-term sheep fetuses exposed to BPA during prenatal development [27].

Evidence of adverse health effects has pushed for restrictions on BPA production in many countries and a gradual withdrawal from the market. As a result, although BPA is still prevalent, other bisphenols have increasingly gained market share [28]. While, at first, BPA replacement could lead to reduced human exposure or exposure to safer compounds, the development of compounds that are structurally or functionally similar raises concerns that substitute chemicals have similar or even stronger adverse effects.

Bisphenol S (BPS) is one of the most commonly used BPA analogs [29], and its structural and physical–chemical similarities to BPA are consistent with its estrogenic-like actions, its binding to other nuclear receptors [30], as well as its neurotoxicity during brain development [29]. To date, few studies have compared the effects of BPA and BPS developmental exposures. However, evidence suggests that despite BPA and BPS similarities, there are neurochemical and behavioral outcomes that differ between them. In this context, the analysis of the expression of genes encoding for elements of the serotonergic system suggests that low-dose perinatal exposure to either BPA or BPS impacts serotonergic neurotransmission in the prefrontal cortex of female rats in the short term, although with gene-specific effects [31]. Regarding the cholinergic system, we failed to identify studies that simultaneously examined the effects of both BPA and BPS in rodents or other mammals. Similarly, when considering studies that only modeled BPS exposure, to our knowledge, none assessed cholinergic markers.

Behavioral analysis that focused on cognitive and mood-related outcomes indicated that perinatal BPA is anxiogenic in male rodents, and increases fear memory and novelty seeking in females, effects that are accompanied by sex-selective imbalance of monoamines and their metabolite levels [25,32,33]. Other types of memory were also shown to be affected when exposure extended from prenatal life to late adolescence, with spatial and recognition memory impaired in both female and male rats and deficient aversive/fear memory identified only in males [34]. As for BPS, although the behavioral outcomes do not always align with those observed in BPA studies, developmental exposure produces measurable effects on several outcomes. Our group has shown that perinatal BPS exposure leads to increased anxiety-like behavior in male rats [35], while exposure restricted to the prenatal period evokes deficits in working and episodic memories in females, increases depressive-like behavior in males, and increases anxiety-like behavior irrespective of the sex [36].

It is increasingly clear that bisphenol compounds act as developmental neurotoxicants [15,16,29] and elevate the risk of cognitive and mood disorders in humans [16,37]. The underlying mechanisms may involve both short- and long-term imbalances in the cholinergic and serotonergic systems. However, to what extent bisphenol exposure disrupts these systems remains largely unexplored. In this regard, comparatively, little is known about compounds other than BPA, such as BPS, and scant studies use both compounds. In this study, we exposed rats to BPA and BPS, aiming to model developmental exposures. Two cholinergic and three serotonergic markers with previously established roles in mood disorders and cognition mechanisms, but poorly explored in models of BPA and BPS exposures, were quantified. The analyses were run in both juveniles and adults, targeting the frontal cortex and pons + medulla oblongata—regions of major serotonergic projections and origins, respectively, and of intense cholinergic activity. We hypothesized that both cholinergic and serotonergic systems would be impacted, although with distinct outcomes depending on whether the animals are exposed to BPA or BPS. Given previous evidence of sex-specific effects in response to bisphenol exposure and of sex-biased prevalence of mood disorders and cognitive deficits, we anticipated that the neurochemical outcomes would vary between females and males.

## 2. Results

Both serotonergic and cholinergic systems were affected by either bisphenol exposure. There were BPA- and BPS-selective effects, and those varied as a function of the dose, the brain region, the sex, and the time point of analysis.

### 2.1. Cholinergic Effects

#### 2.1.1. Short-Term (PN21)

**Figure 1:**
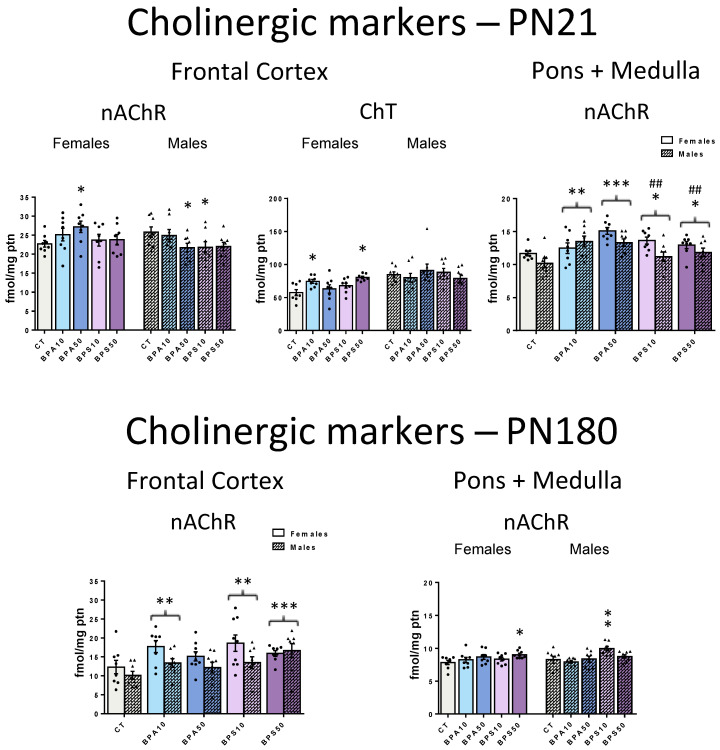
α4β2 nicotinic acetylcholine receptor (nAChR) and high-affinity presynaptic choline transporter (ChT) binding in the frontal cerebral cortex and pons + medulla oblongata of male and female rats whose dams were exposed to BPA, BPS, or vehicle during the gestation and lactation periods. Top panels show data from juvenile rats, collected at the end of the exposure period (PN21), while bottom panels show data obtained long after its end, at adulthood (PN180). CT, control progeny; BPA10, progeny exposed to 10 μg/kg/day of bisphenol A; BPA50, progeny exposed to 50 μg/kg/day of bisphenol A; BPS10, progeny exposed to 10 μg/kg/day of bisphenol S; BPS50, progeny exposed to 50 μg/kg/day of bisphenol S. Values are means ± SEM. * *p* < 0.05, ** *p* < 0.01, *** *p* < 0.001 vs. CT; ## *p* < 0.01 vs. BPA50. Whenever sex-dependent effects were identified, significant pairwise comparisons between groups were shown on the left for females, and on the right for males. Otherwise, female and male bars of a given group were placed side-by-side, and brackets indicate that this group, when collapsed by sex, differs from another group.

By the end of the period of developmental exposure, the analysis of the frontal cortex cholinergic system (Figure 1, top left and middle panels) revealed sex-dependent effects on the regulation of the nAChR (Treatment × Sex − F_4,70_ = 2.5, *p* = 0.053) and the ChT (Treatment × Sex − F_4,70_ = 2.7, *p* = 0.035). Separate analyses for females and males pointed to a female-only increase in ChT binding that reached significance in response to the low dose of BPA (BPA10 > CT, +29.4%) and the high dose of BPS (BPS50 > CT, +39.8%). nAChR binding was increased in female rats exposed to the high dose of BPA (BPA50 > CT, +19.8%). Distinctively, in males, nAChR binding was decreased as a result of exposure to the high dose of BPA (BPA50 < CT, −15.7%) and the low dose of BPS (BPS10 < CT, −15.4%).

In the pons + medulla oblongata (Figure 1, top right panel), BPA and BPS increased nAChR binding in both males and females (Treatment − F_4,70_ = 6.9, *p* < 0.001), an effect that reached significance even at the low dose (BPA10, +18.6%; BPA50, +29.9%; BPS10, +13.4%; BPS50, +13.2%) when compared to CT rats. ChT was not affected (Supplementary Materials Figure S1).

#### 2.1.2. Long-Term (PN180)

Early exposure to endocrine-disrupting chemicals may lead to health issues that either persist or manifest later in life. Here, long-term effects were evident in both the frontal cortex and pons + medulla oblongata. In the frontal cortex (Figure 1, bottom left panel), there were no longer sex-dependent effects. Both bisphenol compounds increased nAChR binding in males and females (Treatment − F_4,75_ = 4.4, *p* = 0.003), with significant effects identified in rats exposed to the low dose of BPA (BPA10 > CT, +38.8%) and to both the low and high doses of BPS (BPS10 > CT, +44.6% and BPS50 > CT, +50.5%). Previous effects identified on the ChT were no longer present at PN180 (Supplementary Materials Figure S1).

In the pons + medulla oblongata (Figure 1, bottom right panel) (Treatment × Sex − F_4,70_ = 2.9, *p* = 0.028), increases in nAChR that were identified by the end of the period of developmental exposure were no longer significant in BPA-exposed rats. However, increased nAChR binding persisted in female rats exposed to the high dose of BPS (BPS50 > CT, +15.4%), and in males exposed to the lower dose of BPS (BPS10 > CT, +20.5%). Neither BPA- nor BPS-exposed rats exhibited significant differences in the ChT when compared to the CT group (Supplementary Materials Figure S1).

### 2.2. Serotonergic Effects

#### 2.2.1. Short-Term (PN21)

**Figure 2:**
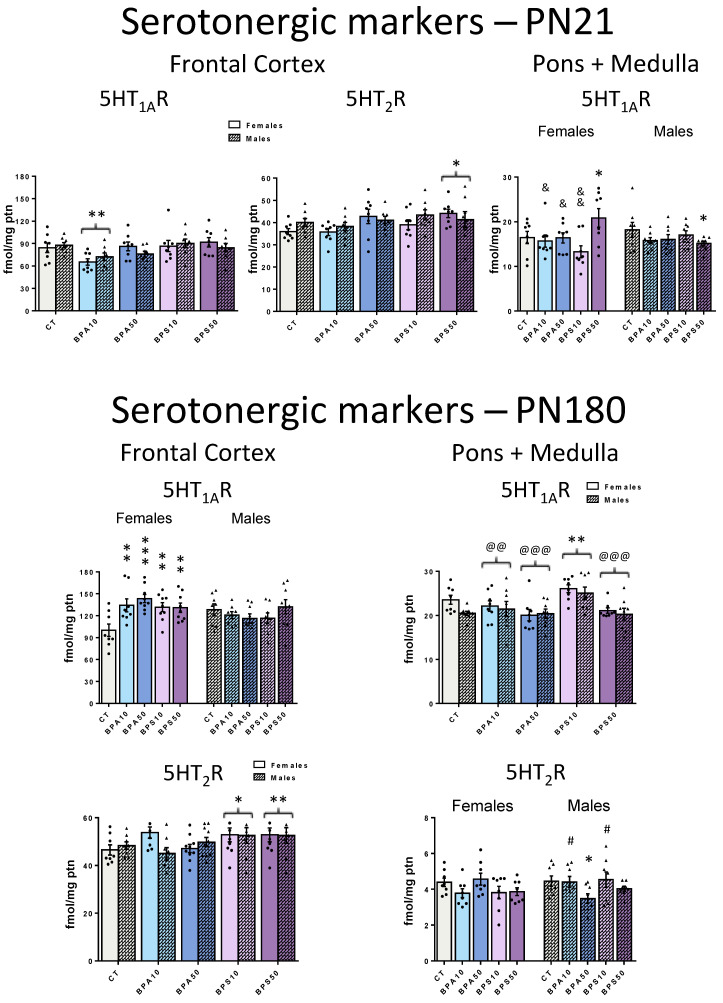
5-HT_1A_ receptor (5-HT_1A_R) and 5-HT_2_ receptor (5-HT_2_R) binding in the frontal cerebral cortex and pons + medulla oblongata of male and female rats whose dams were exposed to BPA, BPS, or vehicle during the gestation and lactation periods. Top panels show data from juvenile rats, collected at the end of the exposure period (PN21), while bottom panels show data obtained long after its end, in adulthood (PN180). CT, control progeny; BPA10, progeny exposed to 10 μg/kg/day of bisphenol A; BPA50, progeny exposed to 50 μg/kg/day of bisphenol A; BPS10, progeny exposed to 10 μg/kg/day of bisphenol S; BPS50, progeny exposed to 50 μg/kg/day of bisphenol S. Values are means ± SEM. * *p* < 0.05, ** *p* < 0.01, *** *p* < 0.001 vs. CT; # *p* < 0.05 vs. BPA50; @@ *p* < 0.01, @@@ *p* < 0.001 vs. BPS10; & *p* < 0.05, && *p* < 0.01 vs. BPS50. Whenever sex-dependent effects were identified, significant pairwise comparisons between groups were shown on the left for females, and on the right for males. Otherwise, female and male bars of a given group were placed side-by-side, and brackets indicate that this group, when collapsed by sex, differs from another group.

The analysis of the serotonergic system revealed that, by the end of the period of developmental exposure, in the frontal cortex (Figure 2, top left and middle panels), both 5-HT_1A_R (Treatment − F_4,70_ = 4.4, *p* = 0.003) and 5-HT_2_R (Treatment − F_4,70_ = 2.5, *p* = 0.049) were affected. 5-HT_1A_R binding was decreased in rats exposed to the low dose of BPA (BPA10 < CT, −20.1%), while BPS increased 5-HT_2_R binding, an effect that reached significance at the higher dose (BPS50 > CT, +12.4%). 5-HTT was not affected (Supplementary Materials Figure S2).

In the pons + medulla oblongata (Figure 2, top right panel), again, only BPS exposure impacted binding: The 5-HT_1A_R (Treatment × Sex − F_4,70_ = 3.7, *p* = 0.008) was increased in females exposed to the high dose (BPS50 > CT, +26.6%) and reduced in high-dose males (BPS50 < CT, −16.8%). Neither BPA nor BPS caused significant changes in 5-HT_2_R and 5-HTT (Supplementary Materials Figure S2).

#### 2.2.2. Long-Term (PN180)

Regarding the frontal cortex serotonergic system (Figure 2, bottom left panels), sex-selective effects emerged in the long term. Both BPA and BPS developmental exposures lead to increased 5-HT_1A_R binding (Treatment × Sex − F_4,75_ = 4.2, *p* = 0.004). These effects were restricted to females and reached significance even at the low dose (BPA10, +34.1%; BPA50, +43.2%; BPS10, +31.5%; BPS50, +30.7%) when compared to CT rats. Early exposure also affected frontal cortex 5-HT_2_R binding (Treatment − F_4,75_ = 3.4, *p* = 0.013). Similarly to the findings at PN21, only BPS significantly caused upregulation of this receptor in adulthood. However, at PN180, the effects were more pronounced, as both the high (BPS50 > CT, +11.2%) and the low (BPS10 > CT, +15.9%) doses resulted in a significant increase in binding.

In the pons + medulla oblongata, the analysis of the serotonergic system (Figure 2, bottom right panels) revealed that both 5-HT_1A_R (Treatment − F_4,70_ = 6.5, *p* < 0.001) and 5-HT_2_R (Treatment × Sex − F_4,70_ = 3.0, *p* = 0.024) were affected in the long term. Similarly to PN21, only BPS exposure impacted 5-HT_1A_R binding. However, in the long term, there were no longer sex-dependent effects: The lower dose of BPS increased 5-HT_1A_R in both males and females (BPS10 > CT, +23.6%). Regarding 5-HT2R, there were late-emergent, sex-dependent effects. 5-HT_2_R binding was decreased, an effect restricted to males early exposed to the higher dose of BPA (BPA50 < CT, −22.0%).

Similarly to PN21, at PN180, there were no effects on 5-HTT both in the frontal cortex and pons + medulla oblongata (Supplementary Materials Figure S2).

## 3. Discussion

To date, few preclinical studies have simultaneously evaluated outcomes of BPA and BPS developmental exposures on neurotransmitter systems relevant to cognitive functions and mood balance. The present study provides evidence that exposure to low doses of BPA or BPS during pregnancy and lactation impairs serotonergic and cholinergic measures in the progeny, with distinct effects depending on the specific compound. Both the frontal cortex and the pons + medulla oblongata are affected, but with region-specific effects. The imbalance in neurotransmitter systems is evident both at the end of the exposure period, in juvenile rats, and long after its end, at adulthood. However, distinct patterns of results emerge depending on when the evaluation is conducted, which aligns with the notion that developmental exposure to these compounds leads to long-term programming effects. Sex-dependent outcomes were also identified but those were not universal, suggesting that mechanisms of action other than or in addition to interference with traditional nuclear or genomic estrogen receptor signaling play a role in the results [8]. Consistent with previous findings on BPA [38] and other endocrine-disrupting chemicals [39], several of the effects were significant at the lower dose, and many of the responses were non-monotonic. Previous studies from our group showed that the rat offspring used in the current study are hyperactive and exhibit increased anxiety-like behaviors in a sex-selective and drug-dependent way [33,35]. Taken together, these data indicate that BPA and its substitute, BPS, exert complex effects on neurodevelopment. The following paragraphs elaborate on these conclusions.

### 3.1. Cholinergic Effects

The α4β2 nAChR was the cholinergic marker more severely affected by early bisphenol exposure. In juveniles, nAChR binding was increased in the pons + medulla oblongata of rats exposed even to the lower dose of BPA and BPS, which poses this brain region as highly sensitive to these compounds’ developmental exposure. Sex-dependent effects were only identified in the frontal cortex, where a very distinct pattern of results was identified for each sex. For females, both nAChR and ChT binding were increased, while, for males, nAChR was decreased. The current data add to previous studies that show that BPA exposure during prenatal development modulates cholinergic-related pathways in the short term [27]. Considering the established role of the cholinergic system in cognitive and mood processes [20,22], these results corroborate the possibility that the disruption of cholinergic signaling plays a role in the development of neurobehavioral problems such as depressed behavior, anxiety disorder, inattention, and delayed language development identified in children exposed to bisphenol during early development [40,41,42].

Long after the end of exposure, at adulthood, the α4β2 nAChR was the only cholinergic marker affected. In the frontal cortex, both BPA and BPS early exposures led to increases in nAChR binding but without sex-dependent effects that were identified in the short term. As for the pons + medulla oblongata, sex-specific effects emerged, and only BPS exposure led to increased nAChR binding. Few studies have investigated whether early bisphenol exposure can program the cholinergic system function. Miyagawa and collaborators [43] showed that adult male mice exposed to BPA during the prenatal and lactation periods exhibit aversive memory impairment and reduced choline acetyltransferase activity, a marker of acetylcholine production, in the hippocampus. The current study adds to Miyagawa’s work by showing that the programming of cholinergic effects by BPA has a broad spectrum, affecting diverse brain regions and components of this neurotransmitter system. Most notably, this is the first study to show that BPS affects the cholinergic system, uncovering new evidence of both long-term and delayed-onset effects.

The α4β2 nAChR, the predominant subtype in the mammalian brain, is differentially expressed across the central nervous system. In rats, its subunits are detectable as early as embryonic day 12, with expression levels progressively increasing through postnatal life until adolescence [44]. Activation of this receptor influences key processes such as neurogenesis, cell differentiation, and synaptogenesis. Accordingly, the impact of the nAChR on brain development seems to depend on its subunits regional and temporal expression [44]. These data are consistent with evidence that the differentiation state of the cell—closely associated with the specific expression of nAChR subunits—can also influence the neurodevelopmental effects of nicotinic agonists [45]. Nicotine, as an nAChR agonist, increases nAChR binding, and this upregulation characterizes a compensatory attempt to correct a state of nicotine-evoked receptor desensitization [46]. For the most part, BPA and BPS increased nAChR binding, mimicking the effects of nicotine. Future studies are warranted to investigate whether the underlying mechanisms are similar to those triggered by nicotine. Of note, the activation of ubiquitously distributed presynaptic nAChRs facilitates the release of several neurotransmitters such as dopamine, noradrenaline, ACh, aspartate, glutamate, and GABA [47,48]. During development, the above-mentioned facilitation of neurotransmitter release mediated by nAChRs is believed to be relevant to the proper maturation of several neurotransmitter systems [44], while, at adulthood, it is believed to be implicated in nAChR-mediated processes such as executive functions that involve attentional and working memory [48,49].

The disruption of the cholinergic system by early BPA and BPS exposures may be mediated through their effects on thyroid function. Both bisphenol compounds disrupt the thyroid system [11,12,30], which regulates neural development, increases cholinergic activity and cognitive function [50,51]. BPA was shown to act as a thyroid hormone antagonist [52]. Supplemental thyroxine increases acetylcholinesterase levels in rats, suggestive of enhanced cholinergic activity [50]. This hormone was shown to reverse scopolamine-induced [50] and nicotine withdrawal-induced [53] deficits in memory/learning, which further points to cholinergic and thyroid signaling interactions. Even short periods of reduced thyroid hormone levels during development can lead to long-lasting effects on cognition [54]. These data are consistent with the possibility that the interference of BPA and BPS in thyroid function contributes to the cholinergic imbalance identified here, leading to cognitive impairment.

### 3.2. Serotonergic Effects

The serotonergic system is involved in a broad spectrum of behaviors and physiological processes, being also implicated in neuropsychiatric disorders. It plays critical roles in the regulation of the sleep–wake cycle and arousal, reward and aversion processing, hedonic responses, expression of learned helplessness, as well as the modulation of mood and cognition [55,56,57]. Among the principal targets of serotonergic projections from the raphe nuclei is the frontal cortex, and both regions are densely populated with 5-HT_1_R and 5-HT_2_R, two of the most abundant and extensively studied subtypes in the brain [58,59]. Accordingly, experimental manipulations of the serotonergic system have been instrumental in elucidating the roles of these receptors, particularly in the regulation of mood [55,60,61,62,63].

Mood disorders are among the most common psychiatric conditions that emerge during childhood [64,65]. Those are often comorbid with cognitive issues such as distorted thinking, attention, judgment, and interpretation biases and linked to altered serotonergic function [19,21,64,65]. Here, both BPA and BPS exposure during the period of pregnancy and lactation altered 5-HT_1A_R and 5-HT_2_R binding in the juvenile progeny. 5-HT_1A_R was decreased in the frontal cortex in response to the lower dose of BPA, while 5-HT_2_R was increased in response to the higher dose of BPS. As for the pons + medulla oblongata, while BPA failed to affect the serotonergic system, the higher dose of BPS evoked sex-selective effects on 5-HT_1A_R: binding was increased in females and decreased in males. These preclinical data raise the possibility that the imbalance of the serotonergic system underlies BPA and BPS effects not only on mood but also on other behaviors as early as in childhood [40,41,42].

At adulthood, there were late-emergent increases in the 5-HT_1A_R binding in the frontal cortex of females exposed to BPA and BPS, while, in the pons + medulla oblongata, increased binding was evident in the BPS progeny irrespective of the sex. As for the 5-HT_2_R, frontal cortical results identified in juveniles were similar to those identified at adulthood, except that at adulthood, binding was increased even at the lower BPS dose. In the pons + medulla oblongata, again, 5-HT_1A_R was increased in both males and females exposed to BPS10. Distinctively, males were targets of a late-emergent decrease in 5-HT_2_R in response to BPA50 early exposure. The identification of both persistent and late-emergent effects of early exposure to BPA and BPS on the serotonergic system suggests multiple mechanisms of action. This possibility is corroborated by the identification of distinct outcomes evoked by BPA and BPS despite their structural similarities. In this regard, although both 5-HT_1A_R and 5-HT_2_R regulate key brain functions, their individual regional distribution and divergent downstream signaling pathways are consistent with evidence that their activation leads to different functional outcomes. The 5-HT_1A_R is highly expressed both as a presynaptic inhibitory autoreceptor on serotonergic neurons in the raphe nuclei, and as a postsynaptic heteroreceptor in serotonergic projecting areas, and there is evidence that the 5-HT_1A_R has distinct signaling repertoires depending on its location (presynaptic or postsynaptic) and the developmental state of the neuron [63]. The 5-HT_2_R activation, in turn, increases extracellular serotonin, supporting an excitatory effect of this receptor on serotonergic neuron activity [59]. However, distinct 5-HT_2_R subtypes are expressed in distinct brain regions, and binding to this receptor has been associated with biased signaling of canonical and non-canonical pathways [66]. Our current data add to previous findings that show short- and long-term increases in 5-HT levels and its turnover in several brain regions in response to BPA prenatal and lactational exposure [67] and in response to a single intracranial injection at PN2 [68].

5-HT neurons express estrogen receptors, and estrogen regulates 5-HT levels by increasing tryptophan hydroxylase 2 expression, thus impacting 5-HT synthesis, and by decreasing monoamine oxidase expression, which results in reduced 5-HT degradation [69,70]. As weak estrogen agonists, both BPA and BPS may disrupt 5-HT neurotransmission by modulating the activity of these enzymes. In this scenario, the altered binding of serotonergic receptors identified in the current study is likely to be secondary to altered 5-HT levels. It is also possible that the serotonergic system imbalance is mediated by BPA and BPS interference with thyroid function [11,12,30]. In this regard, thyroxine was shown to affect the 5-HT system by increasing 5-HT_2A_R expression [71], while triiodothyronine supplementation desensitizes both pre- and postsynaptic 5-HT_1A_R [72] and, in a mouse model of Alzheimer’s disease, reduces this receptor expression [73].

### 3.3. Sex Differences

It is becoming increasingly clear that sex is a crucial variable in the investigation of the effects of environmental contaminants [74]. Despite that, this parameter is often neglected in basic research [75], and the contribution of sex differences to the pathophysiology of environmental neurotoxicants remains an important knowledge gap. Even though the underlying mechanisms responsible for the cholinergic and serotonergic differences between males and females exposed to BPA and BPS are not clear, sex-selective actions of these compounds may be associated with their potential actions as endocrine disruptors. Sex differences in early emergence—even prior to the adolescent sex steroid surge—indicate that BPA and BPS early exposures alter the cholinergic and serotonergic neurocircuitry during its initial course of formation. In this regard, the period of BPA and BPS exposure coincides with the classic perinatal period of steroid-dependent organizational events in the brain [76]. In addition, the current evidence of developmental programming of the adult neural function, which is consistent with previous findings [33,35], points to BPA- and BPS-mediated altered developmental trajectories of neural circuits. In most cases, sex differences attributed to bisphenol exposure are discussed in light of these compounds interactions with steroid and non-steroid hormones [33,35]; however, recent evidence points to a role of sex chromosomes beyond that of hormonal effects. In this context, BPA has been shown to exert epigenetic effects such as altering DNA methylation, thereby affecting neuronal development [77]. Assessment of these and other mechanisms possibly associated with cholinergic and/or serotonergic systems’ effects should be amenable to further investigation and may provide a clearer picture of the role of these systems in the outcomes of BPA and BPS developmental exposure.

The current study includes the assessment of two serotonergic receptors and one subtype of nAChR. Although those are the most abundant serotonergic and cholinergic receptors in the brain, with established roles in mood disorders and cognition mechanisms [20,22,63,66,78,79,80], the quantification of other serotonergic and cholinergic receptors, as well as of components of intracellular signaling cascades, would contribute to a better understanding of the impact of developmental bisphenol exposure on neurotransmission. Another limitation is the lack of BPA and BPS quantification in the offspring. Although estimated intake values in fetuses and infants [4,81,82] suggest that our doses are not far from those of environmental exposures, measuring levels in milk and serum would be valuable to directly relate the administered dose to the actual contamination in the offspring and to compare these levels with those observed in humans.

## 4. Materials and Methods

### 4.1. Animals and Treatment (Figure 3)

All procedures were approved by the Institute of Biology/UERJ Ethical Committee for Animal Research (protocol #: CEUA/016/2017, approved on March 28, 2017), minimizing the number of animals used and avoiding animal suffering, and in accordance with the Brazilian Law no. 11.794/2008. Wistar rats were housed in a temperature-controlled room (23 ± 1 °C) with artificial dark–light cycles (lights on at 7:00 a.m., lights off at 7:00 p.m.), with free access to water and standard chow (Nuvilab^®^ CR1, Sogorb Inc., São Paulo, Brazil–3.36 Kcal/g: 63% carbohydrates, 26% protein, and 11% lipids, in %Kcal). All animals were housed in bisphenol-free polysulfone (PSU) cages. All material and consumables used in their maintenance were also bisphenol-free to avoid contamination.

**Figure 3:**
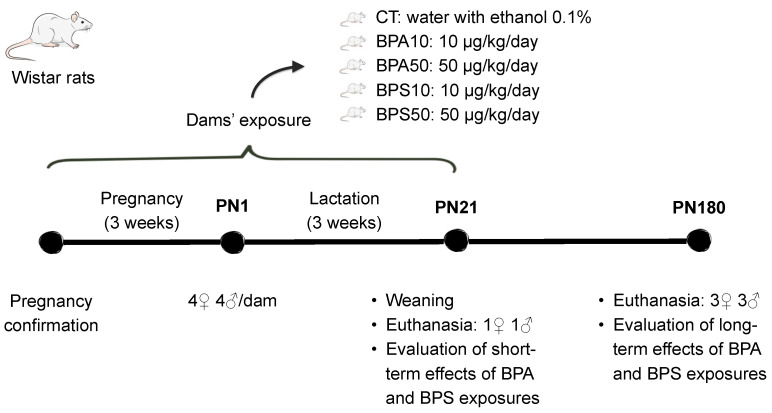
Timeline of the experiment. PN, postnatal day. Groups: CT, control progeny; BPA10, progeny exposed to 10 μg/kg/day of bisphenol A; BPA50, progeny exposed to 50 μg/kg/day of bisphenol A; BPS10, progeny exposed to 10 μg/kg/day of bisphenol S; BPS50, progeny exposed to 50 μg/kg/day of bisphenol S. Exposure (intragastric gavage) was carried out during pregnancy and lactation. After birth, litters were culled to four males and four females. One male and one female were euthanized at PN21, while the remaining animals were euthanized at PN180. Cholinergic and serotonergic markers were evaluated at both time points to assess short- and long-term effects.

Three-month-old, nulliparous female rats were mated with male rats (2:1) for one week. Pregnancy was confirmed by the identification of spermatozoa under an optical microscope (gestational day 1). Pregnant dams were housed singly and randomly assigned into one of five groups: a control (CT) group composed of dams (n = 9) that received water with 0.1% ethanol; two Bisphenol A groups composed of dams that either received 10 μg/kg/day (BPA10, n = 9) or 50 μg/kg/day (BPA50, n = 10) of Bisphenol A diluted in 0.1% ethanol; and two Bisphenol S groups composed of dams that either received 10 μg/kg/day (BPS10, n = 9) or 50 μg/kg/day (BPS50, n = 9) of Bisphenol S. Treatment fluids were administered by gavage every morning (once daily) during the gestation and breastfeeding periods (Figure 3). Of note, the dose of ethanol, used as a diluent of BPA and BPS, was below 1 µg/kg of body mass and, for the purposes of this study, considered biologically negligible.

In humans, the first 1000 days of life is a critical period for the proper maturation of organs, including the brain [83]. The prenatal and lactation phases in the rat may serve as a comparable critical period, during which there is a high sensitivity to environmental agents [84]. These periods are characterized by neurodevelopmental processes that include proliferation, migration, differentiation, synaptogenesis, myelination, and apoptosis, making the brain particularly vulnerable to disruption [84]. BPA and BPS doses were chosen based on risk assessment studies. The U.S. Environmental Protection Agency (EPA) and the U.S. Food and Drug Administration (FDA) established a BPA safe dose of 50 μg/kg/day [85], whereas, recently, the European Food Safety Authority (EFSA) estimated a much lower tolerable daily intake (TDI) of 0.2 ng/kg/day, which is inferior to estimated doses of dietary exposure to BPA [86]. Regarding BPS, the EPA report in 2015 proposed a no-observed-adverse-effect level (NOAEL) of 10 mg/kg/day for systemic effects [87]. EFSA, in turn, defined a NOAEL of 60 mg/kg/day for systemic toxicity and 20 mg/kg/day for developmental toxicity [88]. Accordingly, the exposure dosages of 10 and 50 μg/kg/day are 200 to 6000× lower than the estimated NOAELs of BPS (10–60 mg/kg). As for BPA, those doses are similar or 5× lower than the EPA and FDA safe dose (50 μg/kg/day). There is limited data on BPS; however, estimated intake doses of BPA suggest that the doses used are not distant from those of environmental exposures. In this regard, the Center for the Evaluation of Risks to Human Reproduction of the US National Toxicology Program estimated that daily intakes of BPA range from 1 to 11 μg/kg of body weight in bottle-fed 0–6-month-old infants and from 1.65 to 13 μg/kg of body weight in 6–12-month-old infants [81]. Geens and collaborators reported estimated intakes from food provided by several national and international agencies, and those ranged from 0.01 to 13 μg/kg/d, with the highest values from children who were bottle- fed [4]. The analysis of fetal liver samples indicated a mean BPA concentration of 2.26 μg/kg, and a reduced capacity of the fetus to metabolize BPA. In this study, individual samples measured as high as 50 μg/kg [82].

At birth, considered as postnatal day 1 (PN1), animals were sexed, and litters were adjusted to eight pups per dam (four females and four males per litter). Cross-fostering was used if necessary to reach the specified litter size (Figure 3). From weaning (PN21) onwards, all offspring received a standard diet for rodents. As reported elsewhere, there were no significant differences in body mass between groups [33,35]. Short- and long-term effects of BPA and BPS exposures were evaluated at PN21 and PN180, respectively. At each time point, one animal of each sex per litter was euthanized (Figure 3). The estrous cycle of the adult female offspring was evaluated every morning from PN150 to PN180: all females showed a regular estrous cycle and were euthanized during diestrous, a phase characterized by small variations in sexual hormones when compared to the other phases of the cycle [89]. All animals were anesthetized with xylazine (Xilazin^®^, 100 mg/kg) and ketamine (Cetamin^®^, 50 mg/kg) and euthanized by cardiac puncture. For each treatment group, sex, and time point, 8 to 10 animals were used. Most adult animals used in this present study were submitted to behavioral tests to evaluate locomotor activity and anxiety-like behavior at PN160 [33,35].

The brain regions were dissected by making blunt cuts through the cerebellar peduncles, whereupon the cerebellum (including flocculi) was lifted from the underlying tissue. The cerebral cortex (forebrain with removal of the hippocampus) was separated from the midbrain  +  pons + medulla oblongata by a cut made rostral to the thalamus. The midbrain was then dissected from the pons + medulla oblongata by making a cut caudal to the inferior colliculus, so that the midbrain contained the entire dorsal raphe nucleus but no descending serotonergic nuclei [90,91]. After tissue dissection, the anterior 1/3 of the right and left cerebral cortices, from now on called frontal cortices, and the pons + medulla oblongata were frozen in liquid nitrogen and stored at −80  °C until assayed.

The frontal cortices and the pons + medulla oblongatas were used for the neurochemical determinations, as they comprise regions of major serotonergic projections and origin, respectively. Three serotonergic biomarkers [the 5-HT_1A_ receptor (5-HT_1A_R), the 5-HT_2_ receptor (5-HT_2_R), and the presynaptic 5-HT transporter (5-HTT)] were assessed. The 5-HT_1A_R and 5-HT_2_R are particularly relevant during the perinatal period due to their role in modulating both neuronal and glial proliferation and maturation [92]. In addition, together with the presynaptic 5-HTT [93], they play major roles in 5-HT-related mental disorders, especially depression and anxiety, and modulate cognition and other functions [63,66,78,80]. Regarding the cholinergic system, we evaluated the α4β2 nicotinic acetylcholine receptor (nAChR) and the high-affinity presynaptic choline transporter (ChT). The α4β2 nAChR is the most abundant cholinergic receptor in the brain, being present in both the frontal cortex and pons + medulla oblongata. The ChT, which transports choline to the presynaptic terminal, is responsive to stimuli that alter cholinergic neuronal activity [94,95]. The cholinergic systems play important roles in several cognitive functions and mood [20,22,79].

### 4.2. Evaluation of Cholinergic and Serotonergic Systems

Tissues were thawed and homogenized in ice-cold 50 mM Tris (pH 7.4) using a homogenizer of the Ultra-Turrax type T10 basic. The homogenate was then sedimented by centrifugation at 39,000× *g* for 15 min. The pellet was resuspended (Ultra-Turrax) in the original volume of buffer, resedimented, and the resultant pellet was resuspended in 1/4 of the original volume using a smooth glass homogenizer fitted with a Teflon pestle. Aliquots of this last resuspension were obtained for measurements of [^3^H]Cytisine binding to nAChRs, [3H]hemicholinium-3 (HC-3) binding to ChT, [3H]8-hydroxy-2-(di-n-propylamino)tetralin to 5-HT_1A_R, [3H]ketanserin to 5-HT_2_R, and [3H]paroxetine binding to 5-HTT, and for membrane protein assessment. Proteins were measured by bicinchoninic acid (BCA) protein assay. All assays have been described in detail in previous papers [91,96,97] and will therefore be presented briefly.

#### 4.2.1. Nicotinic Acetylcholine Receptor and Choline Transporter

[^3^H]Cytisine binding to nAChRs was assessed at a final concentration of 1 nM [^3^H]cytisine in the membrane fraction. Incubations were conducted for 75 min at 4 °C in a buffer consisting of 250 μL of 120 mM NaCl, 5 mM KCl, 2.5 mM CaCl_2_, 1 mM MgCl_2_, and 50 mM Tris HCl (pH 7.4). Nonspecific binding was assessed by displacement with 20 μM of nicotine. The binding of [3H]HC-3 to ChT was determined using a final ligand concentration of 2 nM in the membrane fraction; incubations lasted 20 min at 20 °C in a buffer consisting of 10 nM NaKHPO4/150 nM NaCl (pH 7.4). Unlabeled HC-3 20 μM was used to displace specific binding.

#### 4.2.2. Serotonin Receptors and Transporter

The 5-HTRs binding were evaluated by using two radioligands: 1 nM [3H]8-hydroxy-2-(di-n-propylamino)tetralin for the 5-HT_1A_R, and 0.4 nM [3H]ketanserin for the 5-HT_2_R. Binding to the presynaptic 5-HTT was evaluated with 85 pM [3H]paroxetine. For the 5-HT_1A_R, incubations lasted for 30 min at 25 °C in a buffer consisting of 50 mM Tris (pH 8), 0.5 mM MgCl_2_, and 0.5 mM sodium ascorbate. For the 5-HT_2_R, incubations lasted 15 min at 37 °C in 50 nM Tris (pH 7.4). For the binding to the presynaptic 5-HTT, incubations lasted for 120 min at 20 °C in a buffer consisting of 50 mM Tris (pH 7.4), 120 mM NaCl, and 5 mM KCl. For all serotonergic markers, 100 μM 5-HT was used to displace specific binding.

All incubations were stopped by the addition of ice-cold incubation buffer, and the labeled membranes were trapped by rapid vacuum filtration onto glass fiber filters (for ChT, 5-HT_1A_R, and 5HTT, filters were presoaked in 0.15% polyethyleneimine). The filters were then washed with incubation buffer, and radiolabeling was determined. Data were obtained by calculating the specific binding per mg of membrane protein. Each measurement obtained in a given brain region of a given animal was considered as the datum in the corresponding statistical analysis.

### 4.3. Materials

Radioisotopically labeled compounds came from PerkinElmer Life Sciences/Revvity (Waltham, MA, USA) or American Radiolabeled Chemicals (Saint Louis, MO, USA): [3H]Cytisine (specific activity 28.7 Ci/mmol), [3H]HC-3 (specific activity 169 and 160 Ci/mmol), [3H]8-hydroxy-2-(di-n-propylamino)tetralin (specific activity 196.5 Ci/mmol), [3H]ketanserin (specific activity 22.8 Ci/mmol), and [3H]paroxetine (specific activity, 24.4 and 20.0 Ci/mmol). Sigma Aldrich (São Paulo, Brazil) was the source for BPA, BPS, bovine albumin, BCA kit, serotonin, polyethyleneimine, trizma hydrochloride, trizma base, and sodium L-ascorbate. VETEC Química Fina Ltd. a (Rio de Janeiro, RJ, Brazil) was the source for all other reagents. The standard diet for rodents was produced by Nuvilab (Sogorb, SP, Brazil). Cetamin^®^ (CEVA, Paulínia, SP, Brazil), and Xilazin^®^ (Syntec, Tamboré, SP, Brazil) were specific for veterinary use.

### 4.4. Statistical Analysis

To reduce the likelihood of type 1 statistical errors that might result from repeated testing of the data set, initially, analyses of variance (ANOVAs) on each variable (nAChR, ChT, 5-HT_1A_R, 5-HT_2_R, and 5-HTT) were carried out. Treatment (CT, BPA10, BPA50, BPS10, and BPS50) and sex were used as between-subjects factors. Significant Treatment effects and Treatment – sex interactions were followed by Fisher’s Protected Least Significant Difference (FPLSD) post hoc tests. Since bisphenols are endocrine disruptors and target sexual hormones, figures showed female and male data in separate bars. When Treatment – sex interactions were identified, to more clearly represent sex-dependent effects, figures displayed significant pairwise comparisons between groups on the left for females, and on the right for males. Distinctively, when there were Treatment effects but no sex interactions, female and male bars of a given group were placed side-by-side, and brackets indicated that pairwise comparisons between groups were significant irrespective of the sex. Supplementary Materials Figures S1 and S2 show biomarker data for which no significant group differences were found. All data were analyzed using the IBM SPSS Statistics for Windows, Version 21.0 (IBM Corp, Armonk, NY, USA.), and compiled as means and standard errors. Significance was assumed at the level of *p* < 0.05 for main effects; however, for interactions at *p* < 0.1, we also examined whether lower-order main effects were detectable after subdivision of the interactive variables [98]. The criterion for interaction terms was not used to assign significance to the effects but rather to identify interactive factors requiring subdivision for lower-order tests of main effects [98]. All raw data are available as Supplementary Materials Sheets S1 to S20.

## 5. Conclusions

During the prenatal and lactation phases, the developing rat brain is highly sensitive to environmental toxicants [84]. Our current set of results is in line with this evidence and points to significant impacts of BPA in the cholinergic and serotonergic systems of the immature brain. Here we further show that its replacement, BPS, affects these same neurotransmitter systems, although with region-, age-, and sex-specific outcomes, which suggests that there is only a partial overlap in the underlying mechanisms involved in each compound’s effects. The results were identified even at low doses and pose both BPA and BPS as neurotoxicants that compromise neurodevelopment and program disorders later in life. These data are consistent with previous studies from our group that showed sex-selective hyperactivity in response to perinatal BPA and BPS, as well as anxiogenic responses associated with BPS exposure, using animals whose brains were analyzed in the current study [33,35]. Considering the increased risk of cognitive deficits and mood disorders associated with these compounds’ developmental exposure [16,37], and the established role of the serotonergic and cholinergic systems on mood and cognition [19,20,21,22], by shedding light on the involvement of BPA and BPS on these neurotransmitter systems’ disruption, the current set of results fills in gaps on the effects of these compounds on the developing brain and raises concerns as to these effects’ impact on brain function.

## Data Availability

All raw data are available as Supplementary Material—Sheets 1 to 20.

## Supplementary Materials

The following supporting information can be downloaded at https://www.mdpi.com/article/10.3390/ijms26199329/s1.

## Abbreviations

The following abbreviations are used in this manuscript:

5-HT	Serotonin
5-HT_1A_R	5-HT_1A_ receptor
5-HT_2_R	5-HT_2_ receptor
5-HTT	Presynaptic 5-HT transporter
ANOVA	Analyses of variance
BCA	Bicinchoninic acid
BPA	Bisphenol A
BPS	Bisphenol S
BPA10	progeny exposed to 10 μg/kg/day of bisphenol A
BPA50	progeny exposed to 50 μg/kg/day of bisphenol A
BPS10	progeny exposed to 10 μg/kg/day of bisphenol S
BPS50	progeny exposed to 50 μg/kg/day of bisphenol S
ChT	High-affinity presynaptic choline transporter
CT	Control progeny
EFSA	European Food Safety Authority
EPA	Environmental Protection Agency
FDA	Food and Drug Administration
FPLSD	Fisher’s Protected Least Significant Difference
HC-3	Hemicholinium-3
nAChR	nicotinic acetylcholine receptor
NOAEL	No-observed-adverse-effect-level
PN	Postnatal day
PSU	Polysulfone
TDI	Tolerable daily intake
