# Supplementary material for: Serotonergic and Cholinergic Imbalance in the Offspring of Rats Exposed to Bisphenol A and Bisphenol S During Pregnancy and Lactation: Short- and Long-Term Effects
Source: Int J Mol Sci. 2025 Sep 24;26(19):9329. doi: 10.3390/ijms26199329 (PMC12525116; doi:10.3390/ijms26199329)
Supplement: Supplementary file 1 [file ijms-26-09329-s001.zip › ijms-3785999 - SM Figures Proof- IJMS.pdf]

Supplementary Figure 1

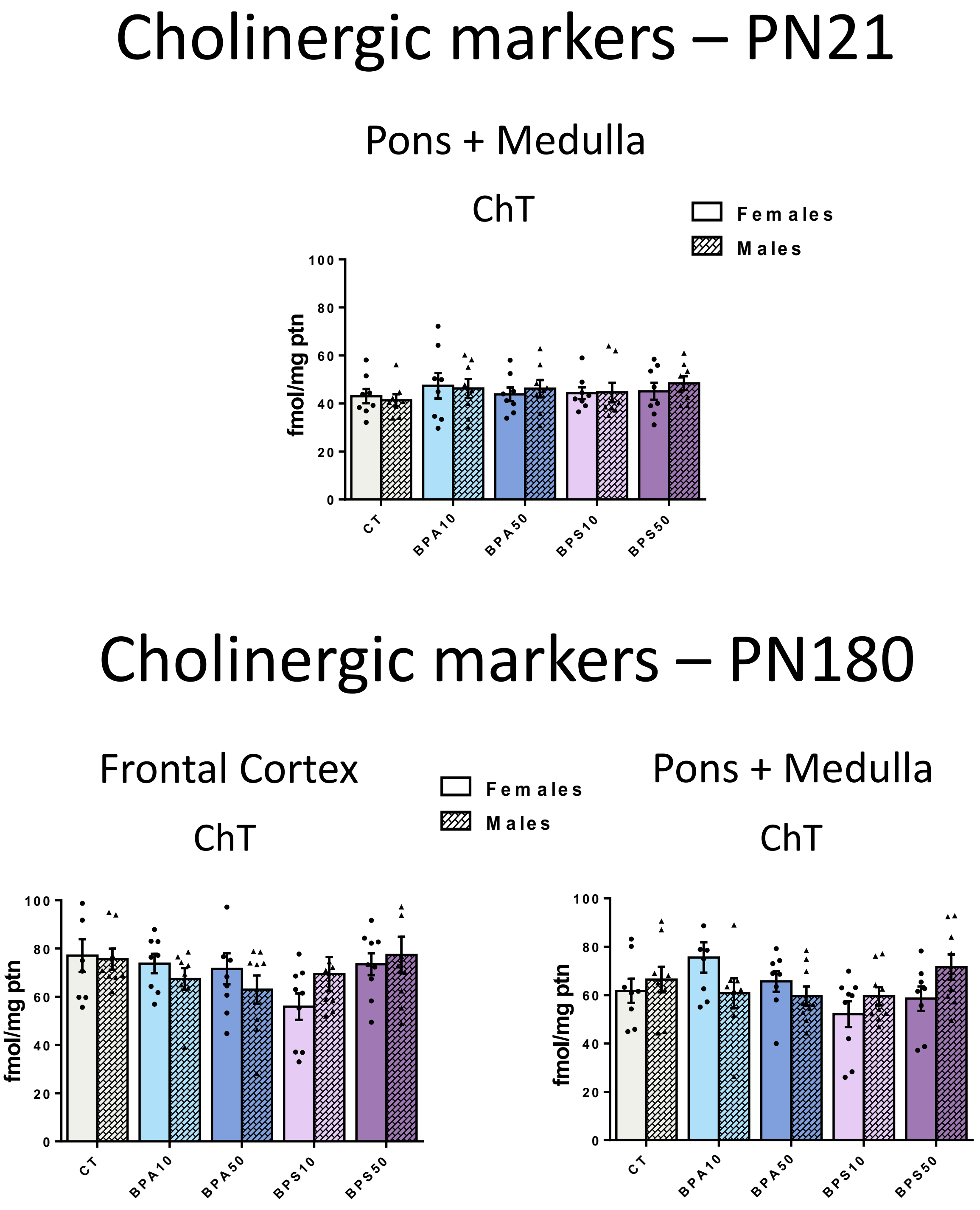

Figure S1. High-affinity presynaptic choline transporter (ChT) binding in the frontal cerebral cortex and pons + medulla oblongata of male and female rats whose dams were exposed to BPA, BPS or vehicle during the gestation and lactation periods. Top panels show data from juvenile rats, collected at the end of the exposure period (PN21), while bottom panels show data obtained long after its end, at adulthood (PN180). CT, control progeny; BPA10, progeny exposed to 10µg/kg/day of bisphenol A; BPA50, progeny exposed to 50µg/kg/day of bisphenol A; BPS10, progeny exposed to 10µg/kg/day of bisphenol S; BPS50, progeny exposed to 50µg/kg/day of bisphenol S. Values are means $\pm$ SEM.

Supplementary Figure 2

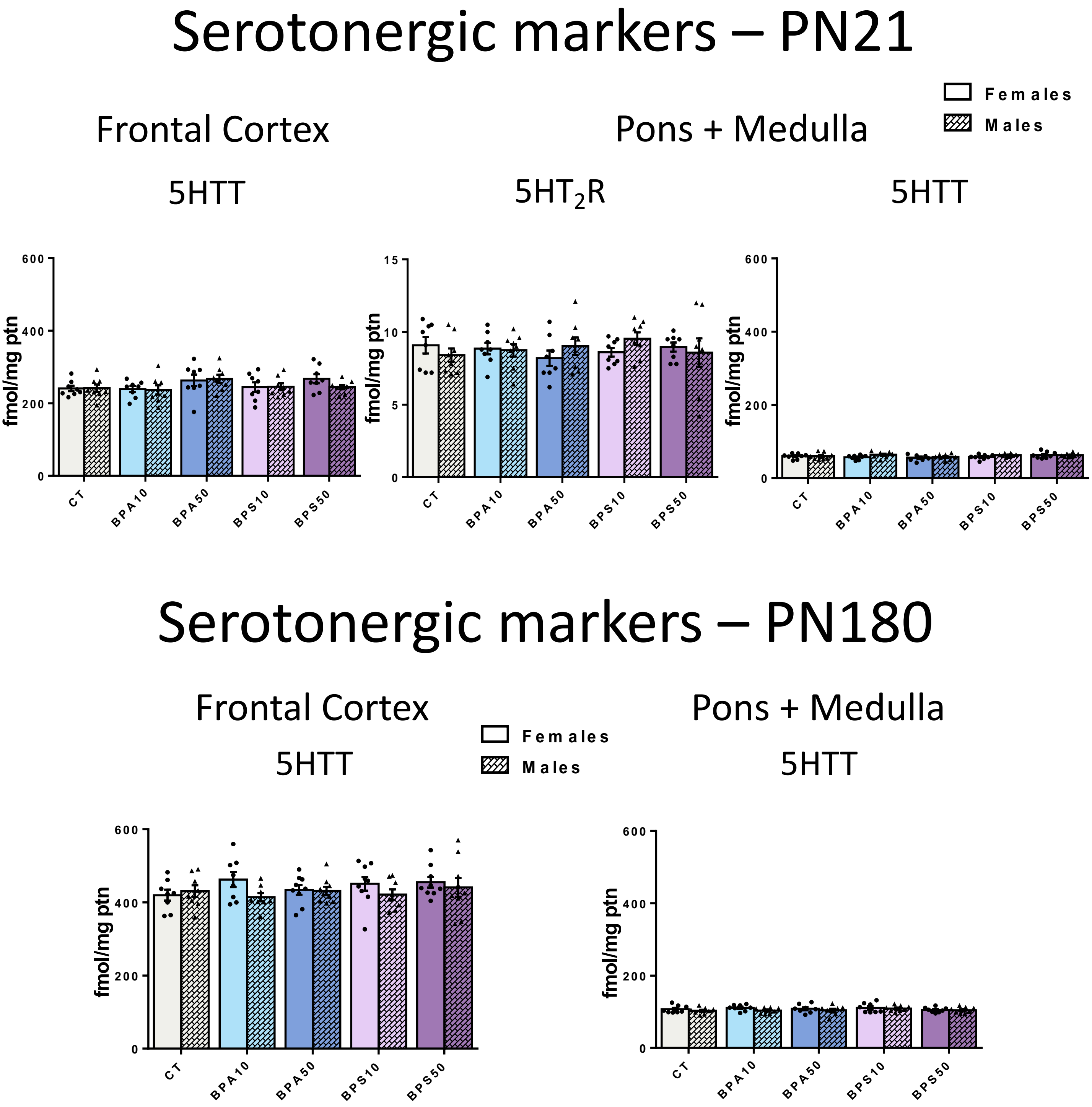

Figure S2. 5-HT_2 receptor (5-HT_2R) and 5HT transporter (5HTT) binding in the frontal cerebral cortex and pons + medulla oblongata of male and female rats whose dams were exposed to BPA, BPS, or vehicle during the gestation and lactation periods. Top panels show data from juvenile rats, collected at the end of the exposure period (PN21), while bottom panels show data obtained long after its end, in adulthood (PN180). CT, control progeny; BPA10, progeny exposed to 10µg/kg/day of bisphenol A; BPA50, progeny exposed to 50µg/kg/day of bisphenol A; BPS10, progeny exposed to 10µg/kg/day of bisphenol S; BPS50, progeny exposed to 50µg/kg/day of bisphenol S. Values are means ± SEM.
